# The Relationship Between Intelligence and Personality Traits Differentiated by Gender in Spanish Adolescents

**DOI:** 10.3390/children12040501

**Published:** 2025-04-14

**Authors:** Ricardo Quintero, Leire Aperribai, Triana Aguirre, Elena Rodríguez-Naveiras, África Borges

**Affiliations:** 1 Department of Clinical Psychology, Psychobiology and Methodology, University of La Laguna, 38200 San Cristóbal de La Laguna, Spain; rquinter@ull.edu.es (R.Q.); taguirre@ull.edu.es (T.A.); naveiras@ull.edu.es (E.R.-N.); aborges@ull.edu.es (Á.B.); 2 Department of Clinical and Health Psychology and Research Methodology, University of the Basque Country UPV/EHU, 20018 Donostia-San Sebastián, Spain

**Keywords:** adolescents, big five, gender, intelligence, personality

## Abstract

Background/Objectives: Intelligence and personality are the most researched constructs in the scientific literature in the field of psychology. Also, the relationship between them has been studied, with controversial results. The present study examines the relationship between intelligence and personality, considering, on the one hand, the influence of gender and, on the other, the predictive capacity of intelligence for personality traits. Methods: The sample consisted of 1166 participants between 11 and 16 years old from the Canary Islands. The Big Five Personality Questionnaire for Children, Adolescents and Adults (MASK-5) and the Adaptive Test of General Intelligence (Matrices-TAI) were used. For the analysis, a quantitative approach with an ex post facto, cross-sectional design was used. The analysis was carried out with SPSS v.26 and Jamovi v.2.3.21. Results: The findings revealed non-significant gender differences in intelligence and personality. The regression model between the two constructs was only significant for the dimension consciousness (*F*(1,1164) = 3.906, *p* = 0.048; *ɳ*_p_^2^ = 0.003) and its facet perseverance (*F*(1,1164) = 5.953, *p* = 0.015; *ɳ*_p_^2^ = 0.005), but the effect size was small in both cases. When considering girls and boys separately, the model was not significant for boys, whereas for girls, the dimension consciousness (*F*(1,595) = 6.148, *p* = 0.013; *ɳ*_p_^2^ = 0.010) and its facet achievement (*F*(1,595) = 8.227, *p* = 0.004; *ɳ*_p_^2^ = 0.014), as well as the facet humility (agreeableness) (*F*(1,595) = 6.472, *p* = 0.011; *ɳ*_p_^2^ = 0.011), were significant, but again, with small effect sizes. Nevertheless, low observed power results for the whole sample and the boys sample suggest the need to increase the sample size. Conclusions: These findings are discussed, and future lines of research in this field of study are proposed.

## 1. Introduction

Since the beginning of the study of individual differences in psychology, research has focused on two essential aspects of individual variability [1,2], intelligence and personality. Both constructs, as specific fields of knowledge, have ended up being among the most researched in the scientific literature. Personality, in one of the original conceptions [3], can be defined as a dynamic organization that determines how people act, think and adjust to their environment. These basic behavioral tendencies, which influence thought, emotions and actions, are of biological origin and develop from childhood to adulthood [4] and can also predict important achievements in life [2]. On the other hand, intelligence can be conceptualized as a general cognitive capacity, which manifests itself as a common factor underlying performance in various cognitive tests [5,6]. Both constructs are characterized by constant and stable attributes [7,8,9,10].

Regarding possible differences between men and women, the debate is still in force after more than a century of research. On the one hand, there is evidence of significant neural differences, both at the structural level [11,12,13] and at the neurofunctional level [14,15], in intelligence and personality. On the other hand, these differences do not imply superiority or inferiority in terms of intelligence [16].

The most recent research suggests that, although there are structural and configurational differences at the brain level between men and women, these are based on biological sex and are minimal, corroborating the theory that both groups present more similarities than differences [17]. On the other hand, it should not be forgotten that there are differences associated with gender roles and, therefore, they are subject to the influences of socialization and the culture in which individuals develop. In this regard, it is worth mentioning some of the most relevant explanatory theories, such as the Cognitive Theory of Social Learning [18], the Sociocultural or Social Role Theory [19] and the Theory of Value of Expectation [20]. Regarding gender, therefore, differences reaching from moderate to significant magnitude are recognized. Some meta-syntheses have summarized the existing research, providing a broader and more detailed view on the subject [17,21]. The present study aims to contribute to determining if there are gender differences related to personality traits and facets in adolescence, since previous research conducted with young adolescents is scarce and has led to controversial results.

On the other hand, the literature on gender differences in cognitive ability has a long and controversial trajectory [22,23,24]. Many meta-analyses have been performed focusing on cognitive ability, specifically on the differential study between verbal ability [25], spatial ability [26], and visuospatial working memory [27], among other constructs. Similarly, studies show gender differences at the attitudinal level. On the one hand, women show greater inhibitory control and perceptual sensitivity in the temperamental plane [28]. In addition, women show greater non-verbal emotional recognition [29] and rumination [30]. On the other hand, men show a greater predisposition to the search for sensations, greater risk-taking and higher self-esteem [31], which has been declining over the years, although still favoring men [32].

A fundamental issue related to gender in the study of individual differences in intelligence has been to try to determine the possible existence of variations in g factor. This question represents a considerable technical challenge, which could explain the lack of attention received in this field of research. The g factor or general intelligence, proposed by Spearman in the early 20th century, refers to the common cognitive ability that underlines all intellectual tasks [6]. According to this theory, intelligence is hierarchically organized, with g at the top of the hierarchy. Likewise, although people may have specific skills in particular areas, there is a general intelligence factor that determines global cognitive ability.

In this regard, some authors have found non-significant differences in either a general sample [33] or young adolescents [34]. These results are consistent with previous research using different test batteries and varied samples in their composition. Developmental evolutionary theories argue that boys and girls experience distinct maturation rhythms, both physically and mentally, throughout childhood and adolescence, which would therefore affect the development of intelligence. Along this line, Lynn et al. [35] found that there were no significant differences in the mean scores between girls and boys for the age group from 6 to 14 years. However, beginning at the age of 15, boys tend to obtain higher scores than girls. This difference becomes more noticeable in adulthood, suggesting that these differences arise only after puberty [35]. The present study aims to ascertain if there are gender differences in general intelligence, mainly based on fluid intelligence, since previous research has led to controversial results based on the confusing conceptualization of intelligence.

Personality is conceptualized as an internal dynamic structure that influences and shapes how individuals interact, think and adapt to their environment. In the field of personality psychology, an essential question is whether a theoretical score for one of the personality traits corresponds to a given behavior. Costa and McCrae [36] presented the Five Factors Model (FFM) and developed a taxonomy that gathers the different personality traits. This theory allows us to explain the functioning of personality as a system formed by five basic tendencies or factors that interact with biological aspects and influences that are external to the formation and maintenance of the adaptive characteristics of the person [4,37].

The FFM organizes traits into five domains or factors: openness to experience, conscientiousness, extraversion, agreeableness, and neuroticism or emotional stability [4,37,38,39,40]. The FFM does not include discrete and independent dimensions but represents a broad level on personality’s hierarchy [41]. However, gender differences can be explored at multiple levels. In this sense, research has predominantly focused on two levels: (1) the general domains of the FFM and (2) a multitude of more specific features, known as facets, which are classified under the FFM. Currently, there is a lack of consensus on the number and exact nature of these facets [42].

From this perspective, differential psychology allows us to elucidate general patterns of behavior in men and women on average, considering that gender differences in terms of average differences do not mean that men and women only experience states at extreme polarities of the trait spectrum. On the contrary, there may be significant differences and even a high degree of overlap between their distributions [43]. In this sense, the study of gender differences in personality through the FFM reveals interesting patterns in behavioral and thought traits.

Openness to experience (O) reflects the trend towards imagination, creativity and intellectual curiosity. This trait does not show significant differences between men and women [44]. Generally, there is divergence between facets, which prevents differences at the domain or trait level [45]. However, evidence supports that women score higher in facets such as esthetics and feelings while men score higher in ideas [46,47]. This difference suggests distinct preferences in approaches within the same trait [48]. Conscientiousness (C) is a trait that is characterized by the tendency to exhibit self-discipline and organization. Women tend to obtain slightly higher scores in facets such as order and self-discipline. However, these differences are not uniform depending on culture and are not always consistently maintained in all aspects of the trait [46,47]. Conscientiousness is the strongest and most consistent personality trait for predicting academic achievement [49].

Extraversion (E) is a trait related to sociability and assertiveness [46]. This feature shows small gender differences [44]. In addition, these differences are dependent on specific facets [45]. Women often obtain higher scores on facets such as warmth, gregarism, and positive emotions, while men excel in assertiveness and the search for emotion [46,47,50].

Agreeableness (A) comprises traits related to altruism, empathy and compassion [46]. It is characterized by an inclination towards collaboration, the promotion of coexistence and respect for the needs of others, in contrast to actions of exploitation or harm to others [45]. Research conducted by Feingold [47] and Lippa [44] reported that women obtain significantly higher scores for agreeableness.

Finally, emotional stability or neuroticism (N) reflects the tendency to experience negative emotions and the processes that arise as a result of the perception of threat and punishment, encompassing aspects such as anxiety, depression, anger, self-awareness and emotional variability [46]. Lippa [44] found moderately higher scores in women, although not always in anger or angry hostility [45]. As a summary, gender differences in FFM vary in magnitude and direction according to specific traits or facets [45]. These differences may reflect a combination of biological, cultural and social factors. Likewise, differences between men and women in personality tend to be higher in egalitarian societies [44,51]. Regarding gender differences in personality during adolescence, there is limited knowledge. There is also a need to study how factors like culture and age influence these differences. The findings report greater neuroticism and conscientiousness in women and more complex patterns in extraversion and agreeableness, which tend to align with those observed in adults around 17 years of age. These differences support that women develop earlier than men, developing certain personality traits earlier, and that this aspect of women’s development is consistent across different cultures [52].

The predictive capacity of intelligence for personality is a topic of special interest in psychology. Although extensive research has been conducted on the relationships and results of cognitive abilities, on the one hand, and personality traits on the other hand, limited knowledge is available on how they are interconnected. Intelligence is not only manifested in skills such as reasoning, but also in adapting to different environments and situations. Understanding this relationship is fundamental, as it provides valuable perspectives in areas such as education and clinical psychology, underlining the role of the relationship between intelligence, personality and human behavior [42,53], which will be explored in this study.

A recent meta-analysis revealed that among the five major personality traits, openness to experience and neuroticism had the most significant associations with intelligence [48]. Moreover, the findings indicated a lack of consistency in the results for extraversion and intelligence, observing that correlations between variables have experienced a change from positive to slightly negative in recent years [54]. It has also been shown that conscientiousness and agreeableness do not show significant correlations with intelligence in general [48,54,55,56].

Personality and intelligence are fundamental traits that affect several factors in life, including academic performance [49], subjective well-being [57,58], health [59,60] and psychopathology [61,62]. Although they have been studied for over a century, the relationship between these two variables remains a mystery, with recent research focusing on understanding how they are interconnected [42,48,55,63]. This study aims to contribute to better understanding this relationship.

### Objectives of the Present Study

The general objectives of this research are the following:

(1) To determine the possible gender differences in intelligence and personality. Based on the scientific literature, two hypotheses are proposed:

**Hypothesis 1.**  *There are no significant gender differences in intelligence* [33,34,35].

**Hypothesis 2.**  *There are no significant gender differences in personality traits* [45,52].

(2) To verify the predictive capacity of intelligence for personality traits. Based on the scientific literature, the following hypothesis is proposed:

**Hypothesis 3.**  *Intelligence has a predictive capacity over the openness and neuroticism personality traits* [48]*, but not over extraversion* [56]*, conscientiousness and agreeableness* [48,54,55,56].

## 2. Materials and Methods

### 2.1. Design

For this study, a survey methodology was conducted, with an ex post facto, cross-sectional design. It is worth noting that this study is part of more extensive research. Specifically, it is part of the project “Women empowerment: Education for Talent Incentive in STEM: EMPODERA” (2020 EDU95).

### 2.2. Participants

A convenience sampling method was used. For this, all secondary-level educational centers of the Autonomous Community of the Canary Islands (Spain) were contacted and those students who had the requested authorizations and who consented participated anonymously in this research during the period of 2021–2022. The sample consisted of 1177 adolescents, of whom 597 (*M*_age_ = 13.13, *SD* = 0.924, range = 11–15) were girls and 569 (*M*_age_ = 13.25, *SD* = 0.987, range = 11–15) boys, and 11 individuals identified their gender as being in the category of “other” (0.9%). In studies where the size and representativeness of the groups are critical factors for validity and statistical relevance, focusing on those segments that offer a more robust and significant perspective is important. Therefore, the latter 11 individuals were excluded from the study, the final sample being composed of 1166 girls and boys. In terms of educational course or year (of the secondary education level), 379 participants (17.4% girls) were in the first year, 426 participants (18.7% girls) in the second year, and 361 participants (14.6% girls) in the third year.

### 2.3. Instruments

The tests used in this study are presented below. All of them are standardized in Spain.

#### 2.3.1. Matrices-TAI: General Intelligence Adaptive Test

Matrices-TAI [64] is a test designed to assess general and fluid intelligence through inductive reasoning tasks with graphic matrices and based on the Item Response Theory (IRT). The instrument can be administered to people aged between 6 and 74 years. This test is adapted to the user’s responses, allowing a personalized and accurate assessment, either individually or collectively, with a duration of approximately 30 min. The scales are adjusted by age and educational level. This instrument comprises 27 items, selected from a total of 326, based on the estimation of the Approval Test Probability (PEA) using Bayesian techniques. First- and second-year students used Level D, whereas third year students used Level E. Data collection is ongoing and complete reliability is not yet achievable. The instrument has shown high reliability (*α* = 0.86) and correlation (*r* = 0.72) in previous studies [64]. Finally, it should be explained that this instrument is based in the Cattell–Horn–Carroll (CHC) theory and measures general intelligence based on fluid intelligence, by applying matrices and removing elements which could be culturally and socially influenced. Therefore, it does not consider crystallized intelligence, which is better influenced by environmental factors.

#### 2.3.2. Mask-5: Big Five Personality Questionnaire for Children, Adolescents and Adults

Mask-5 [65] is a self-report test to assess personality traits which analyzes the five major personality factors (openness to experience, conscientiousness, extraversion, agreeableness, and neuroticism or emotional stability) and their respective facets. MASK-5 consists of a total of 164 items and 25 scales (five per dimension). In addition, it is applied in a wide variety of age groups: adolescents and adults over 12 years of age. The complete version of the test is available in the editorial (TEA Ediciones), and provides a detailed evaluation of all relevant variables and facets. The estimated time to complete this version is approximately 30 min. The authors report good psychometric properties, including high reliability with internal consistency coefficients ranging between 0.85 and 0.92. In addition, the exploratory factor analysis supports the structure of the questionnaire, finding correlations in the hypothesized sense. The instrument is in preparation for marketing in TEA Ediciones [66].

### 2.4. Procedure

First of all, this research obtained the authorization of the Ethics Committee of Research and Animal Welfare of the University of La Laguna (Spain), meeting the ethical requirements of the research and obtaining authorization to carry out the study according to the proposed terms (CEWOULD 2021-0449). Secondly, the educational centers of the Autonomous Community of the Canary Islands were contacted to request authorization to carry out the research. After obtaining authorization, the students and their families received an informative report so that they could be aware of the research. Informed consent was requested, in accordance with the regulations for conducting tests and confidentiality of data. Once the authorizations were obtained, the tests were administered in the educational centers, collectively and in the presence of researchers and faculty, lasting two and a half hours. At the beginning of the tests, the students were informed about the confidentiality of the data, and they were also assigned an individual code to maintain their anonymity. The instruments used were completed in computerized format through the online assessment platform of TEA Ediciones (TEAcorrige).

### 2.5. Data Analyses

Initially, a process of reliability analysis of the intelligence and personality tests was carried out. Then, to determine if there were statistically significant differences in intelligence assessments between the two groups (girls and boys), a Student *t* test for independent samples was performed. After that, in order to examine the discrepancies between gender and multiple personality dimensions, a Multivariate Analysis of Variance (MANOVA) was carried out. This analysis allowed us to determine whether the independent variables had a significant impact on the dependent variable. Finally, a general linear model (GLM) multivariate regression analysis was performed to analyze possible relationships between intelligence and personality traits. The analyses were carried out with SPSS v.26 and the Jamovi program, version 2.3.21.

## 3. Results

According to the objectives of this study, the obtained results are presented in three main sections: (1) Intelligence and gender; (2) Personality and gender; and (3) Intelligence’s capacity to predict personality.

### 3.1. Intelligence and Gender

To determine the existence of statistically significant differences in general intelligence between the two groups, girls and boys, a Student *t* test for independent samples was performed. As it is the prescriptive standard test, a homogeneity test of variances was performed. The null hypothesis of the assumption of equality of variances was rejected (*F*(1,1164) = 5.64, *p* < 0.05), so the Welch test for the contrast of the differences in means between both groups was performed. With a result of *t*(1139) = −0.591, *p* = 0.555, *d* = −0.03, it was concluded that there are no significant differences in intelligence between boys (*M* = 100; *SD* = 15.8) and girls (*M* = 101; *SD* = 14.2).

### 3.2. Personality and Gender

Firstly, reliability analyses were performed, both for the questionnaire as a whole and for each factor separately. The results indicate that the internal consistency [67] was good for the whole scale (α = 0.84) and for all dimensions (with α values between 0.84 and 0.89), except for agreeableness, which was acceptable (α = 0.77) (see Table 1). These results highlight the reliability of the instrument used.

Secondly, to determine whether variations in the independent variable (gender) exert a significant influence on the dependent variables (personality traits), and to assess the level of relationship between them, a MANOVA analysis was performed after determining the homogeneity of variances (χ^2^ (15) = 12.9; *p* = 0.609). The analysis did not show significant differences (λ Wilks = 0.992; *F*(5,1160) = 1.81; *p* = 0.109).

### 3.3. Intelligence’s Capacity to Predict Personality

To ascertain the predictive capacity of intelligence in each of the personality dimensions, a multivariate regression analysis was performed between intelligence (as a covariable) and the five personality traits and their facets (as dependent variables). Considering the whole sample (see Table 2), only the conscientiousness trait showed a significant value (*F*(1,1164) = 3.906, *p* = 0.048; partial *ɳ*^2^ = 0.003), and within this dimension, so did the perseverance facet (*F*(1,1164) = 5.953, *p* = 0.015; partial *ɳ*^2^ = 0.005), but the effect sizes were small for both.

When considering the girls subsample (see Table 3), only the conscientiousness trait showed a significant value (*F*(1,595) = 6.148, *p* = 0.013; partial *ɳ*^2^ = 0.010), and within this dimension, so did the achievement motivation facet (*F*(1,595) = 8.227, *p* = 0.004; partial *ɳ*^2^ = 0.014), as well as the humility facet (*F*(1,595) = 6.472, *p* = 0.011; partial *ɳ*^2^ = 0.011) of the agreeableness dimension. Also, the effect sizes for girls were small in all cases.

As for the boys subsample (see Table 4), all values remained non-significant and the effect sizes were small (partial *ɳ*^2^ < 0.06).

## 4. Discussion

Personality and intelligence are broad and fundamental spheres of individual differences. It is important to highlight that gender similarities and differences in this regard are influenced by a variety of social, cultural and biological factors. In addition, it should be remembered that these constructs directly influence different contexts, such as academic, work and social contexts.

Considering all of the above, this study aims to clarify the type of relationship between gender, intelligence and personality, focusing on two fundamental questions: Firstly, to what extent are there gender differences in intelligence and personality? And secondly, to what extent can general intelligence predict personality traits? On the one hand, regarding the differences between boys and girls in general intelligence (g factor) and personality (traits), the results of this study do not show significant differences, confirming the first and second hypotheses of this study. Both results are consistent with those found in the scientific literature [33,34,44,47,68]. Therefore, a direct implication for educational and clinical spheres would be the recommendation of avoiding gender bias by also considering the assessment of fluid intelligence, since in our study, gender differences did not appear. On the other hand, regarding the capacity of intelligence to predict personality, the results of the multivariate regression indicate that general intelligence alone cannot effectively predict personality traits, contradicting the third hypothesis. Nevertheless, in a meta-analysis carried out by Anglim et al. [48], the relationship between personality and intelligence was analyzed. Only the openness dimension of personality showed a positive significant correlation when intelligence was measured by matrices, with this being a weak correlation. The same happened with the negative significant correlation shown with the neuroticism dimension. In the same meta-analysis, they considered the mean age of participants as a moderator variable, and when this was under 18, similar results were found with the openness and neuroticism dimensions. Also, for the conscientiousness dimension, a weak correlation was found. When considering gender as a moderator, when the females sample was between 25% and 75% (as in this study), the openness, neuroticism, and conscientiousness dimensions showed significant but weak correlations. This meta-analysis ends by concluding that the relationship between personality and intelligence is more nuanced than implied by the big five domains. Therefore, they propose a better understanding at the facet level. Our study also considers facet-level results, indicating a weak capacity of intelligence to predict personality facets.

Given the nature of the sample, it should be taken into account that adolescence is a critical period of development, which suggests that the results could vary significantly in different stages of life. Furthermore, the results of this study show slight differences in girls but no differences in boys, an aspect that may be related to developmental reasons. These results may not be in accordance with the relationships between the two variables found in recent studies [42,48]; according to Anglim et al. [48], at a mean age of greater than 60, correlations of general intelligence increased significantly from weak to medium for openness and neuroticism.

Limitations of the study: This study has a limitation that means we should consider the results with caution. Specifically, the use of probabilistic sampling, instead of convenience sampling, could be more representative of the adolescent population. Also, even though in this case, a large sample was used (*N* = 1166), representing a considerable number of the adolescents of the Autonomous Community of the Canary Islands, the use of a larger sample to analyze intelligence’s capacity to predict personality would contribute to improving the results. Nevertheless, it is important to underline that personality has not been largely studied in adolescent populations, and that this study contributes to clarifying its relationship with general intelligence. It should be mentioned that there is little evidence of personality traits and facets in this developmental stage; Anglim et al. [48] mentioned only 10 studies (out of the 278 studies in their meta-analysis) with participants aged between 11 and 15 years, in which only 4 found that non-verbal intelligence was related to personality traits, but none related to personality facets. Also, it would be interesting to conduct a study using probabilistic sampling that examines differences or similarities in the development of intelligence and personality between genders throughout adolescence. This would be important to verify the developmental theory, focusing specifically on how different maturation processes in men and women can influence the evolution of certain psychological characteristics [52,69]. In addition, it could be interesting to adopt an item approach, as recommended by different authors [70,71]. This would allow a more detailed analysis of the results, as it is a model that represents a broad level of personality hierarchy, and in which factors cannot be considered to be discrete and totally independent dimensions [41]. Finally, considering crystallized intelligence could also be interesting to better understand how it is related to personality traits, facets and items, when considering gender as a moderating variable.

Understanding and delimiting the determinants of human behavior is a complex task. In this research, attention was focused on two of its main elements, intelligence and personality. However, both constructs interact very closely with other variables, among them self-esteem, perfectionism and coping strategies, so the exploration of such interaction undoubtedly represents a particularly attractive line of research, especially from a multidimensional approach. It is important to examine the implications of these trends in the educational and clinical fields, since both intelligence and personality have the potential to significantly influence a variety of fundamental outcomes in life. On the one hand, to consider fluid intelligence would permit girls to detect cognitive capacities out of biases related to environmental factors and to intervene for greater academic performance [49]. On the other hand, understanding how personality traits and facets develop in adolescence and adult age would help when applying psychological interventions directed toward vocational interests [44], subjective well-being [57,58], health [59,60] and psychopathology [61,62].

## 5. Conclusions

In conclusion, this study has contributed to the body of literature stating that there are no significant differences between girls and boys in terms of general intelligence (fluid intelligence) and personality traits (big-five dimensions). The study has also highlighted intelligence’s lack of capacity to predict personality traits and facets in adolescence. Therefore, considering, on the one hand, the relevance of both intelligence and personality in the optimal development of an individual as well as in their adjustment in everyday life, in multiple contexts, and, on the other hand, the social influences/expectations linked to gender, it is essential to continue investigating their interaction and how these dimensions mutually influence development throughout life. In particular, it is crucial to examine how gender norms and expectations can shape or limit the expression of intelligence and personality traits and facets in different social, educational and cultural contexts and at which developmental stage these expressions would be manifested.

## Figures and Tables

**Table 1:** Means, standard deviations and reliability indices of personality factors.

Dimension	Mean (Standard Deviation)			Cronbach’s Alpha
	Girls (*n* = 597)	Boys (*n* = 569)	General Sample	General Sample
O	86.4 (15.4)	85.1 (15.6)	85.7 (15.5)	0.831
C	88.2 (15.0)	87.9 (15.4)	88.0 (15.2)	0.881
E	85.4 (15.7)	86.4 (15.4)	85.9 (15.5)	0.849
A	89.2 (12.3)	87.4 (13.0)	88.3 (12.7)	0.773
N	76.4 (16.6)	78.0 (16.3)	77.2 (16.5)	0.841

**Table 2:** The results of the multivariate regression analysis between intelligence and personality for the whole sample (*N* = 1166).

Dimension	Facet	*F*	*df*	*p*	*ɳ* *_p_* ^2^
Openness		1.545	1,1164	0.214	0.001
	Imagination	2.334	1,1164	0.127	0.002
	Curiosity	0.373	1,1164	0.541	0.000
	Originality	1.128	1,1164	0.288	0.001
	Esthetic sensitivity	0.930	1,1164	0.335	0.001
	Self-observation	0.432	1,1164	0.511	0.000
Conscientiousness		3.906	1,1164	0.048	0.003
	Organization	2.539	1,1164	0.111	0.002
	Formality	0.724	1,1164	0.395	0.001
	Achievement motivation	3.033	1,1164	0.082	0.003
	Perseverance	5.953	1,1164	0.015	0.005
	Self-efficacy	1.964	1,1164	0.161	0.002
Extraversion		0.315	1,1164	0.575	0.000
	Joviality	0.613	1,1164	0.434	0.001
	Sociability	1.085	1,1164	0.298	0.001
	Optimism	0.219	1,1164	0.640	0.000
	Vitality	18.494	1,1164	0.259	0.001
	Self-worth	1.450	1,1164	0.229	0.001
Agreeableness		0.954	1,1164	0.329	0.001
	Empathy	0.397	1,1164	0.529	0.000
	Honesty	0.766	1,1164	0.382	0.001
	Goodness	0.104	1,1164	0.747	0.000
	Humility	1.433	1,1164	0.231	0.001
	Altruism	1.315	1,1164	0.252	0.001
Neuroticism		0.038	1,1164	0.845	0.000
	Calm	0.420	1,1164	0.517	0.000
	Depressive mood	0.314	1,1164	0.575	0.000
	Frustration tolerance	0.182	1,1164	0.670	0.000
	Resilience	0.352	1,1164	0.553	0.000
	Self-acceptance	0.168	1,1164	0.682	0.000

**Table 3:** The results of the multivariate regression analysis between intelligence and personality in girls (*n* = 597).

Dimension	Facet	*F*	*df*	*p*	*ɳ* *_p_* ^2^
Openness		2.046	1,595	0.153	0.003
	Imagination	1.686	1,595	0.195	0.003
	Curiosity	0.482	1,595	0.488	0.001
	Originality	3.769	1,595	0.053	0.006
	Esthetic sensitivity	0.869	1,595	0.352	0.001
	Self-observation	0.462	1,595	0.497	0.001
Conscientiousness		6.148	1,595	0.013	0.010
	Organization	3.091	1,595	0.079	0.005
	Formality	2.945	1,595	0.087	0.005
	Achievement motivation	8.227	1,595	0.004	0.014
	Perseverance	3.834	1,595	0.051	0.006
	Self-efficacy	3.298	1,595	0.070	0.006
Extraversion		0.098	1,595	0.755	0.000
	Joviality	0.041	1,595	0.840	0.000
	Sociability	0.844	1,595	0.359	0.001
	Optimism	0.106	1,595	0.745	0.000
	Vitality	0.531	1,595	0.466	0.001
	Self-worth	0.997	1,595	0.318	0.002
Agreeableness		3.173	1,595	0.075	0.005
	Empathy	1.449	1,595	0.229	0.002
	Honesty	1.598	1,595	0.207	0.003
	Goodness	0.070	1,595	0.791	0.000
	Humility	6.472	1,595	0.011	0.011
	Altruism	2.710	1,595	0.100	0.005
Neuroticism		0.082	1,595	0.775	0.000
	Calm	1.046	1,595	0.307	0.002
	Depressive mood	0.201	1,595	0.654	0.000
	Frustration tolerance	0.706	1,595	0.401	0.001
	Resilience	0.990	1,595	0.320	0.002
	Self-acceptance	0.980	1,595	0.323	0.002

**Table 4:** The results of the multivariate regression analysis between intelligence and personality in boys (*n* = 569).

Dimension	Facet	*F*	*df*	*p*	*ɳ* *_p_* ^2^
Openness		0.117	1,567	0.733	0.000
	Imagination	0.739	1,567	0.390	0.001
	Curiosity	0.019	1,567	0.892	0.000
	Originality	0.155	1,567	0.694	0.000
	Esthetic sensitivity	0.178	1,567	0.673	0.000
	Self-observation	0.065	1,567	0.799	0.000
Conscientiousness		0.179	1,567	0.672	0.000
	Organization	0.276	1,567	0.600	0.000
	Formality	0.191	1,567	0.662	0.000
	Achievement motivation	0.063	1,567	0.803	0.000
	Perseverance	2.238	1,567	0.135	0.004
	Self-efficacy	0.072	1,567	0.789	0.000
Extraversion		0.203	1,567	0.653	0.000
	Joviality	0.778	1,567	0.378	0.001
	Sociability	0.331	1,567	0.566	0.001
	Optimism	0.091	1,567	0.763	0.000
	Vitality	0.702	1,567	0.402	0.001
	Self-worth	0.586	1,567	0.444	0.001
Agreeableness		0.045	1,567	0.833	0.000
	Empathy	0.036	1,567	0.850	0.000
	Honesty	0.009	1,567	0.926	0.000
	Goodness	0.028	1,567	0.868	0.000
	Humility	0.386	1,567	0.535	0.001
	Altruism	0.020	1,567	0.886	0.000
Neuroticism		0.002	1,567	0.968	0.000
	Calm	0.016	1,567	0.900	0.000
	Depressive mood	0.147	1,567	0.701	0.000
	Frustration tolerance	0.042	1,567	0.837	0.000
	Resilience	0.012	1,567	0.912	0.000
	Self-acceptance	0.133	1,567	0.716	0.000

## Data Availability

Data will be available on request, by contacting the corresponding author.
